# Mapping research trends in obsessive-compulsive disorder before and after the COVID-19 pandemic: a bibliometric analysis focusing on its molecular mechanisms

**DOI:** 10.3389/fpsyt.2025.1615497

**Published:** 2025-07-02

**Authors:** Yuito Inoue, Nobutoshi Ichise, Wataru Ukai, Jun Shinozaki, Toshifumi Ogawa, Takuro Karaushi, Marenao Tanaka, Yukinori Akiyama, Masato Furuhashi, Atsushi Kuno, Tatsuya Sato

**Affiliations:** ^1^ Department of Cellular Physiology and Signal Transduction, Sapporo Medical University School of Medicine, Sapporo, Japan; ^2^ Department of Neuropsychiatry, Sapporo Medical University School of Medicine, Sapporo, Japan; ^3^ Department of Institutional Research, Center for Medical Education, Sapporo Medical University, Sapporo, Japan; ^4^ Department of Systems Neuroscience, Sapporo Medical University School of Medicine, Sapporo, Japan; ^5^ Department of Cardiovascular, Renal, and Metabolic Medicine, Sapporo Medical University School of Medicine, Sapporo, Japan; ^6^ Tanaka Medical Clinic, Yoichi, Japan; ^7^ Department of Department of Neurosurgery, Sapporo Medical University, School of Medicine, Sapporo, Japan; ^8^ Department of Pharmacology, Sapporo Medical University School of Medicine, Sapporo, Japan

**Keywords:** obsessive-compulsive disorder (OCD), bibliometric analysis, VOSviewer, COVID-19, molecular-focused research

## Abstract

Obsessive-compulsive disorder (OCD) is a psychiatric disorder that primarily develops during adolescence, and is characterized by obsessive thoughts and compulsive behaviors. Although multiple factors including heredity, environment, and abnormalities in neural networks and synapses are involved in the onset and exacerbation of OCD, their underlying molecular mechanisms have not been fully elucidated. In addition, recent studies have demonstrated that the novel coronavirus disease (COVID-19) pandemic worsened OCD phenotypes. Hence, this global crisis may have changed the field of molecular-focused OCD research. We conducted a brief bibliometric analysis to investigate changes in prevalent topics in molecular-focused OCD research before (2015-2019) and after (2020-2025) the COVID-19 pandemic using Web of Science and VOSviewer. “Schizophrenia” and “metaanalysis” remained highly ranked terms in molecular-focused OCD research. In terms of neurotransmitters, the term “serotonin” became more prevalent than “dopamine” after the COVID-19 pandemic. In addition, research interest shifted toward younger populations, and there was a noticeable increase in terms related to neural networks such as “connectivity”. However, only a few specific molecular mechanisms or cellular physiological pathways by which COVID-19 exacerbates OCD have been identified. To address this gap, an additional *post hoc* analysis focusing on inflammation-related terms was conducted, revealing the emergence of “oxidative stress” and “c-reactive protein” in studies published after the COVID-19 pandemic. The findings of this study highlight several potential clues for elucidating the pathophysiology of OCD and identifying aggravating factors such as COVID-19, while also emphasizing the importance of continued molecular-focused research to establish novel therapeutic targets.

## Introduction

Obsessive-compulsive disorder (OCD) is a mental disorder that primarily develops in adolescence and young adulthood, and is characterized by obsessive thoughts and compulsive behaviors (1). Its estimated prevalence in the general population ranges from approximately 1-3%, although the degree of severity varies (2). The mechanisms underlying OCD development are thought to be complex, and genetic and environmental factors may modify its course (3). However, the cellular and molecular pathophysiology of OCD remain unclear.

Several neurobiological mechanisms, such as specific brain circuits, have been identified as central to understanding the pathophysiology of OCD. For example, it has been suggested that OCD is characterized by structural abnormalities and changes in functional connectivity in neural circuits centered on the prefrontal cortex and the striatum, and that it shows overactivity of the reward system and a decrease in cognitive control (4). In light of the remarkable recent progress in molecularly targeted therapies in various fields (5–7), research on the paradigm shift toward molecularly targeted therapies and personalized treatments for OCD is urgently needed.

The novel coronavirus disease (COVID-19) has dramatically affected people’s lives and influenced various health conditions. Although the general consequences of the COVID-19 pandemic on mental health are clear (8), there has been particular interest in its potential effects on OCD. As OCD may interact with pandemic-related fears, and given substantial symptom heterogeneity (9), it is important to determine whether particular OCD clinical characteristics are associated with worse mental health outcomes. Concerns about harm to oneself or others and a heightened propensity toward disgust may result in excessive washing and cleaning behaviors in many individuals with OCD (10, 11). These behaviors, along with a fear of contamination, are among the most prevalent OCD symptoms (12). OCD is also associated with an intolerance of uncertainty and an inflated sense of responsibility to prevent harm (13). As the COVID-19 pandemic created heightened sense of responsibility for safety, increased need for cleaning behaviors, and considerable uncertainty, individuals with OCD may have faced a higher risk of adverse mental health effects. Indeed, the COVID-19 pandemic has been associated with worsening OCD symptoms (14–17), as well as relapse from previously well-controlled OCD (18, 19). However, beyond recognizing OCD phenotypes influenced by the COVID-19 pandemic, underlying molecular mechanisms by which the COVID-19 pandemic causes OCD deterioration remains unclear. In addition, post-pandemic shifts in key topics in the field of molecular-focused OCD research remain unknown. Clarifying these changes may provide valuable insights into therapeutic approaches and research priorities, particularly in the post-COVID-19 pandemic era.

Bibliometric analysis is increasingly recognized as a valuable approach to obtain important insights into recent research trends and identifies the frontiers of research, and this evaluation of globally recognized published papers identifies the frontiers of research in a given filed (20). Therefore, this brief research study conducted a bibliometric analysis of published papers investigating OCD and its molecular-focused factors. We identified relevant trends in studies from the five years before the COVID-19 pandemic (2015–2019) and from the pandemic to the present (2020-2025).

## Methods

### Bibliometric analysis

Data were extracted using Web of Science, and data processing was performed using VOSviewer (21, 22). To investigate research trends and extract keywords, regarding molecular-focused research, we used two search terms “OCD (obsessive-compulsive disorder)” and “molecular (All Fields)”. We selected the “Article” filter to limit the search results to research papers. The search results were analyzed using VOSviewer to identify their primary research topics. The analysis was conducted for research papers published before and after the COVID-19 pandemic. First, search results from 2015 to 2019 (before the COVID-19 pandemic) were considered. Second, search results from 2020 to 2025 (after the COVID-19 pandemic) were analyzed. A flow chart of the bibliometric analysis is shown in **Supplementary Figure S1**.

To further explore topic-specific trends that may not have been fully captured in the initial Web of Science-based analysis, we conducted a *post hoc* VOSviewer analysis using titles and abstracts retrieved from the PubMed database. This analysis targeted the term “inflammation” based on the context of the COVID-19 pandemic. Searches were conducted using the following query syntax in PubMed: (“obsessive-compulsive disorder” OR “OCD”) AND “inflammation” AND (“2015/01/01”[Date - Publication]: “2019/12/31”[Date - Publication]) or (“2020/01/01”[Date - Publication]: “2025/02/26”[Date - Publication]). Retrieved records were exported and analyzed with VOSviewer.

## Results

### Publication trends from January 1994 to February 2025, considering search results obtained using terms “ OCD (obsessive-compulsive disorder) “ and “molecular”

**Figure 1:**
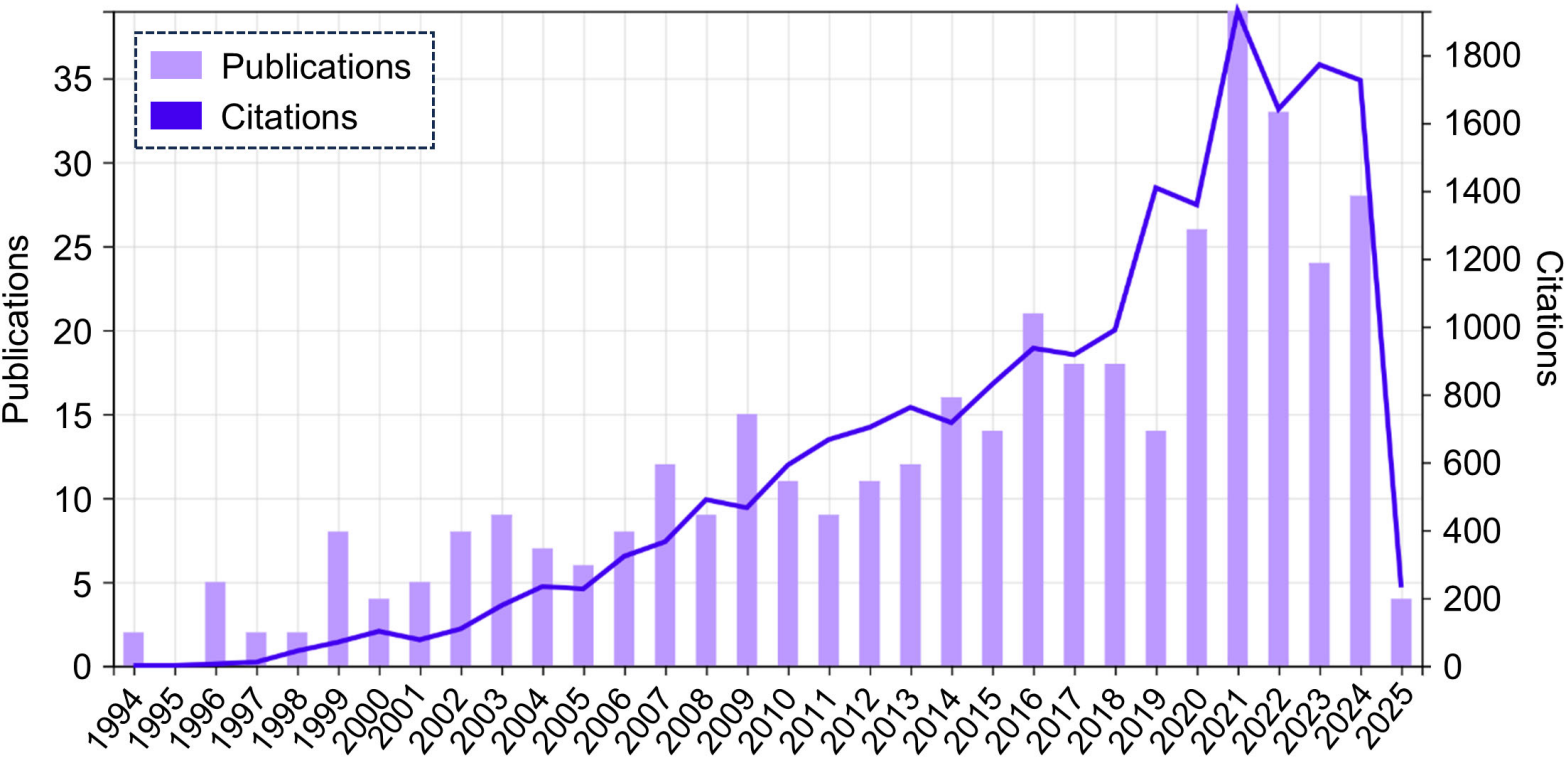
Trends in publications and citations on the molecular aspects of OCD research (January 1994 - February 2025). This figure illustrates the annual trends in publications (purple bars) and citations (blue line) related to molecular research on OCD from 1994 to 2025. The year 2025 included data from January 1, 2025, to February 26, 2025. OCD, Obsessive-compulsive disorder.

Trends in the number of publications and citations related to OCD and molecular-focused research since 1994 are shown in **Figure 1**. The number of publications and citations has generally increased over time, but these increases leveled off in 2021. The number of publications has been decreasing since then, whereas the number of citations has remained nearly constant. These findings suggest that the COVID-19 pandemic has had a direct or indirect impact on research on OCD and its underlying molecular mechanisms.

### Mapping the trends in OCD research focusing on the molecular mechanisms before and after the COVID-19 pandemic

**Figure 2:**
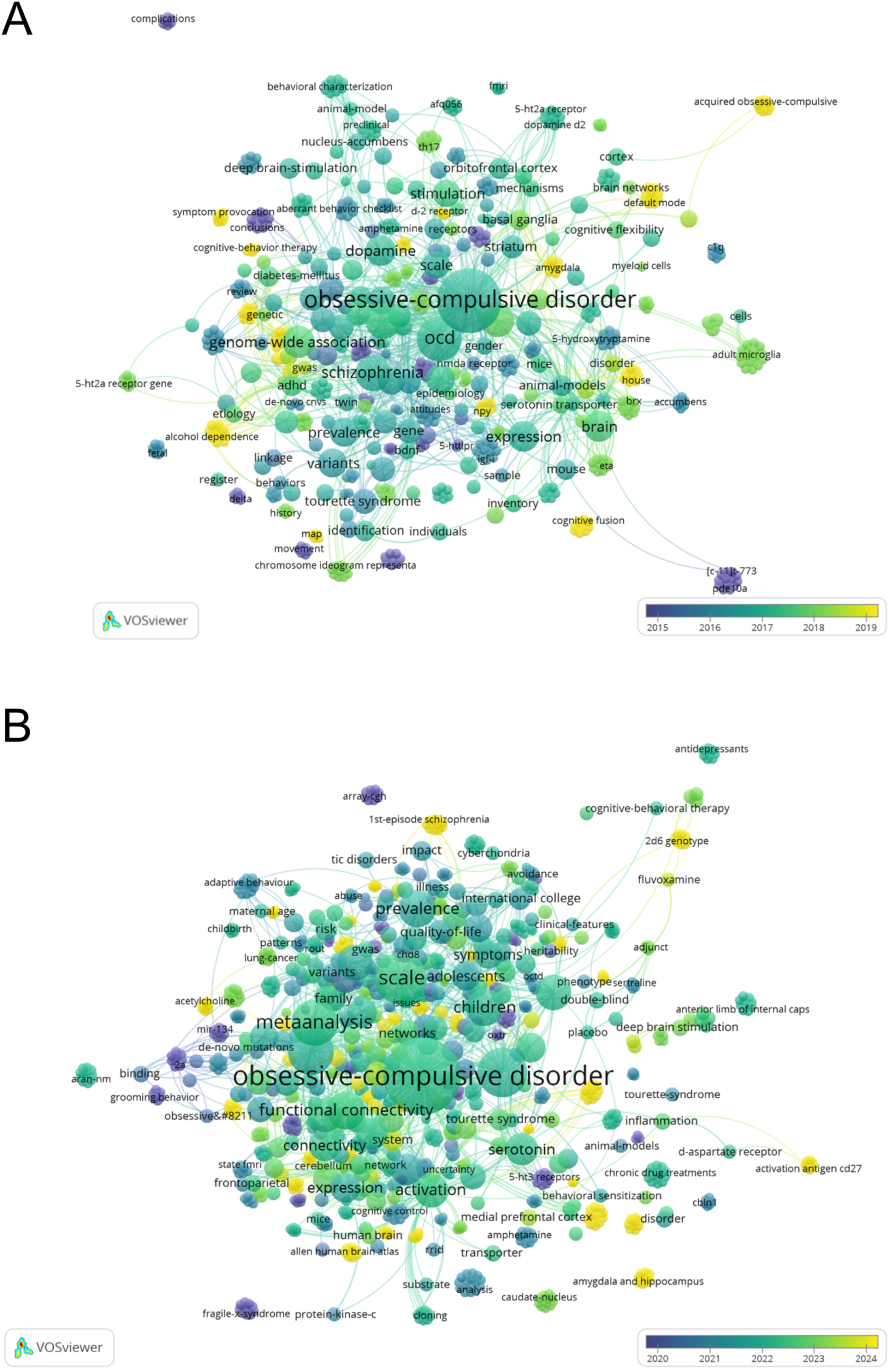
Comparison of bibliometric analysis networks in molecular-focused OCD research (2015–2019 vs. 2020–2025). These networks were visualized using VOSviewer. The figures illustrate the overlay visualization of the keyword co-occurrence in molecular-focused OCD research across two periods: 2015–2019 **(A)** and 2020–2025 **(B)**. The year 2025 included data from January 1, 2025, to February 26, 2025. The nodes represent frequently occurring keywords with larger nodes indicating a higher frequency. Links represent co-occurrences with stronger connections, as indicated by the thicker lines. Color gradients indicate the average publication year of each keyword with earlier years shown in blue and more recent years shown in yellow-green. OCD, Obsessive-compulsive disorder.

To explore changes in critical research topics over time and their correlations, maps of research topics targeting on the molecular-focused OCD research from 2015 to 2025 are shown in **Figure 2**. Before the COVID-19 pandemic (2015–2019), the terms “schizophrenia” and “genome-wide association” were prevalent, appearing as large circles in the center of the VOSviewer map (**Figure 2A**). In addition, “dopamine” was also clearly identified as a key term related to neurotransmitters in molecular-focused OCD research (**Figure 2A**). In contrast, from 2020 to 2025, after the COVID-19 pandemic, the term “serotonin” gained prominence (**Figure 2B**). In addition, terms related to younger populations such as “children” and “adolescents” emerged during this period (**Figure 2B**). Furthermore, brain functional terms, including “functional connectivity”, “activation”, and “medial prefrontal cortex” were prominent. As anticipated, the term “inflammation” appeared as a ley research topic after the COVID-19 pandemic; however, it was not located at the center of the VOSviewer map. Although an overreaction of the immune system due to COVID-19 infection has been associated with the worsening of OCD symptoms (23), it remains unclear whether studies during the COVID-19 pandemic specifically focused on the relationship between molecular-focused OCD research and inflammation.

### Comparing the 30 most prevalent topics in molecular-focused OCD research before and after the COVID-19 pandemic

To identify the changes in research topic prevalence before and after the COVID-19 pandemic, we ranked terms according to their prevalence in molecular-focused OCD research before and after the COVID-19 pandemic. The 30 most prevalent terms are presented in **Table 1**. From 2015 to 2019, in addition to the terms “OCD” or “obsessive-compulsive disorder”, the terms such as “schizophrenia”, “expression”, “genome-wide association”, “dopamine” and “metaanalysis” ranked highly. This indicates that pre-pandemic research primarily focused on gene expression, neural circuits, and neurotransmitters, with particular emphasis on dopamine. From 2020 to 2025, “metaanalysis” and “schizophrenia” remained highly prevalent, indicating that these terms are becoming increasingly critical areas of focus. Whereas the term “striatum” ranked withing the 30 most prevalent terms before the COVID-19 pandemic, “basal ganglia” gained increased attention after the pandemic. These changes suggest that an increased emphasis on integrating research through meta-analyses, exploring the associations between OCD and other psychiatric disorders, and evaluating the pathology and severity of OCD following the COVID-19 pandemic. The prominence of “basal ganglia” also suggests a growing interest in this brain region’s role as a crucial neural circuit in OCD.

**Table 1:** Trends in key terms in molecular-focused OCD research before and after the COVID19 pandemic.

2015-2019 (before the COVID19 pandemic)				2020-2025 (after the COVID19 pandemic)			
Rank	Keyword	Total link strength	Occurences	Rank	Keyword	Total link strength	Occurences
1	obsessive-compulsive disorder	39	495	1	obsessive-compulsive disorder	60	704
2	ocd	19	256	2	ocd	25	306
3	schizophrenia	12	152	3	scale	28	275
4	mataanalysis	12	149	4	metaanalysis	22	252
5	brain	9	124	5	schizophrenia	21	235
6	anxiety	10	123	6	functional connectivity	15	175
7	expression	9	120	7	obsessive compulsive disorder	13	157
8	dopamine	9	114	8	children	14	156
9	genome-wide association	10	110	9	prevalence	14	151
10	children	7	103	10	brain	12	138
11	depression	8	102	11	association	13	136
12	prevalence	8	89	12	activation	12	121
13	genetics	6	79	13	serotonin	11	118
14	bipolar disorder	5	78	14	connectivity	11	113
15	polymorphism	6	75	15	depression	9	113
16	stimulation	7	75	16	anxiety	9	106
17	comorbidity	7	74	17	basal ganglia	10	106
18	scale	7	74	18	abnormalities	10	102
19	variants	8	74	19	adolescents	8	99
20	adolescents	5	71	20	expression	9	94
21	symptom dimensions	6	71	21	genome-wide association	8	93
22	risk	6	68	22	comorbidity	8	90
23	tourette syndrome	6	68	23	dysfunction	8	89
24	association	5	67	24	neurobiology	7	89
25	protein	6	64	25	cortex	8	86
26	neurobiology	5	62	26	prefrontal cortex	7	85
27	striatum	5	61	27	gene	7	84
28	adhd	4	60	28	stimulation	7	84
29	behavior	6	60	29	symptoms	8	84
30	gene	6	60	30	severity	6	81

### A *post hoc* analysis of trends in inflammatory targets in OCD Research before and after the COVID-19 pandemic

**Figure 3:**
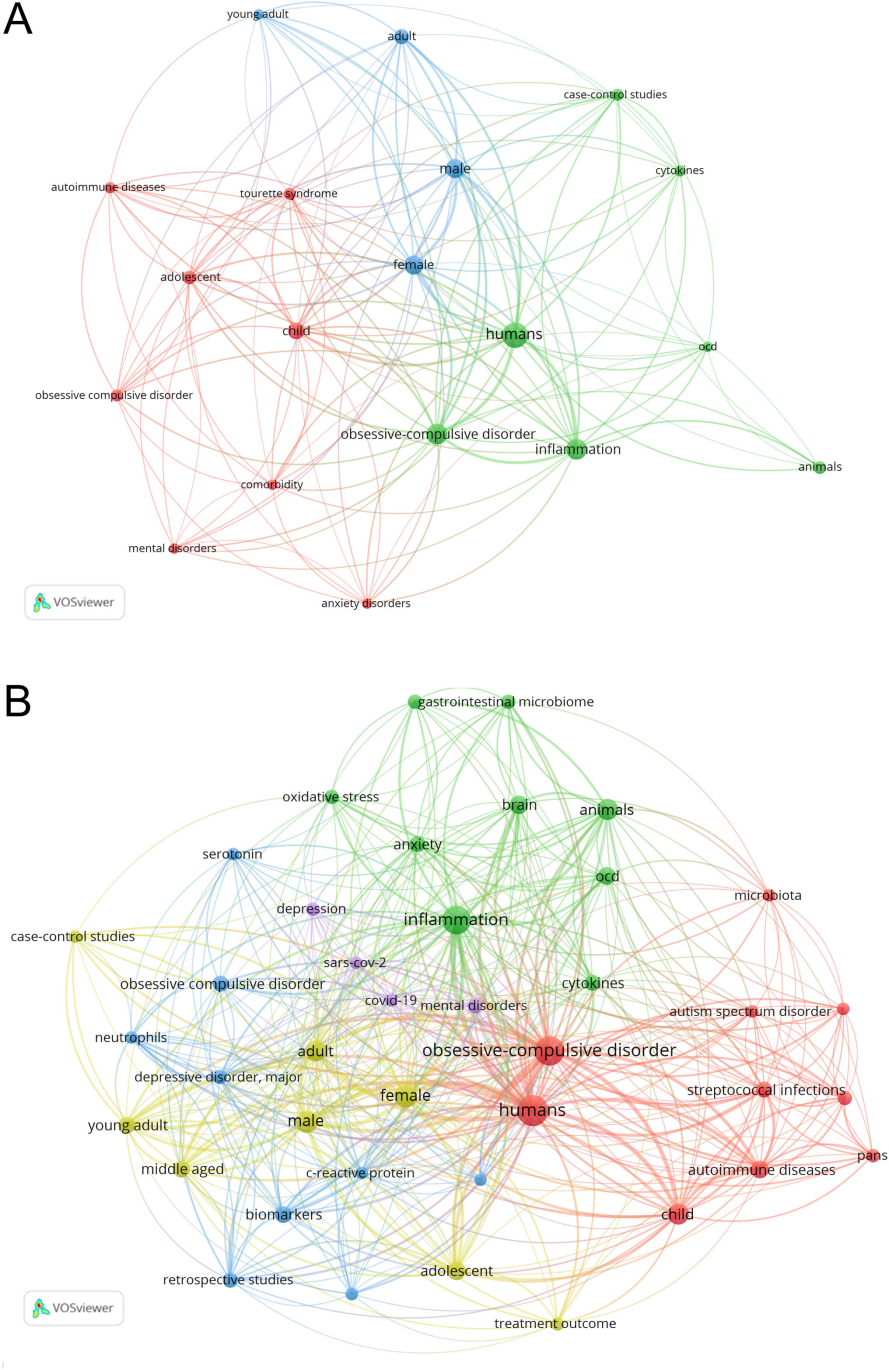
*Post hoc* visualization of keywords related to inflammation in OCD Research (2015–2019 vs. 2020–2025). These networks were visualized using VOSviewer as part of a *post hoc* analysis to further examine molecular-related keywords in OCD research. The figures illustrate the network visualization of the keyword co-occurrence with “inflammation” in OCD-related literature across two periods: 2015–2019 **(A)** and 2020–2025 **(B)**. The year 2025 included data from January 1, 2025, to February 26, 2025. The nodes represent frequently occurring keywords with larger nodes indicating higher frequency. Links represent co-occurrences, with stronger connections, as indicated by the thicker lines. Colors indicate clusters of closely related terms based on co-occurrence strength. OCD, Obsessive-compulsive disorder.

Since the initial bibliometric analyses did not identify the pathophysiological relationship between molecular-focused OCD research and the COVID-19 pandemic, an additional *post hoc* analysis was conducted focusing on the term “inflammation”, which is particularly relevant in the context of COVID-19 (**Figure 3**). Few notable molecular-focused terms were observed before the pandemic (**Figure 3A**); however, several molecular-related terms such as “oxidative stress” and “c-reactive protein” as well as “cytokines” were identified as frequent terms after the pandemic (**Figure 3B**). These results suggest a growing research interest in specific molecular targets, particularly those related to inflammation, in the context of OCD and COVID-19 pandemic.

## Discussion

This present study revealed an upward trend in molecular-focused OCD research, which reached its peak in 2021. Although the causal relationship between worsening of OCD phenotypes and the COVID-19 pandemic remains unclear, there was a qualitative change in the most prevalent research topics before and after the COVID-19 pandemic. Although “COVID-19” did not rank within the 30 most prevalent terms in molecular-focused OCD research after the COVID-19 pandemic, “inflammation”, which is related to COVID-19, did. This suggests that the COVID-19 pandemic may have had some impact on molecular-focused OCD research. In fact, a *post hoc* analysis focusing on inflammation identified the terms “oxidative stress”, “c-reactive protein” and “cytokines” in studies published after the COVID-19 pandemic, suggesting that these factors may have become emerging topics of interest in OCD research. Evidence from recent reviews suggests that oxidative stress and inflammation may contribute to the pathophysiology of OCD by disrupting the tryptophan-kynurenine pathway (24), while ketogenic diets, with their anti-inflammatory and antioxidant properties, have been proposed as a promising metabolic intervention for modulating these underlying mechanisms (25). In addition, research on PANDAS (Pediatric Autoimmune Neuropsychiatric Disorders Associated with Streptococcal infections), but not COVID-19 itself, may have affected the results (26), although the inhibition of inflammation has been proposed as a novel therapeutic target for OCD (27). Interestingly, Saini et al. reported three cases of individuals who developed both OCD and arthritis following COVID-19 infection (28), suggesting that certain biological responses to COVID-19 including systemic inflammation may be directly or indirectly involved in the development of OCD. Similar to the proposed mechanism for PANDAS, activation of the immune system may be an important pathway in the pathophysiology of OCD.

Bipolar disorder and Tourette’s syndrome were ranked as overlapping symptoms of OCD among the 30 most prevalent terms before the COVID-19 pandemic. According to a previous study, 10% of patients with OCD also have bipolar disorder (29). Tourette’s syndrome is a hereditary disorder characterized by vocal and motor tics and is associated with compulsive traits (30). Before and after the COVID-19 pandemic, the two keywords of “metaanalysis” and “schizophrenia” continued to rank highly in prevalence. This indicates that their importance has remained consistent and that there is growing interest in comprehensive reviews and the relationship between OCD and other overlapping mental disorders including schizophrenia. Indeed, OCD and schizophrenia share certain environmental and neurobiological factors, and 12-25% of patients with schizophrenia develop OCD (31). While a single case report has described the simultaneous onset of schizophrenia and OCD (32), it is important to distinguish between schizophrenia and OCD in molecular-focused OCD research. Although this analysis identified a strong focus on gene expression in OCD research, few keywords were found for proteins – that is, those translated from genes-, or for their post-translational modifications and supermolecular structures which are important for protein function and physiological roles (33, 34). Given the recent advances in proteomics and omics analyses of protein modifications (35, 36), these new technologies may provide new approaches for elucidating the pathophysiology of OCD.

This study also demonstrated that the term “children” ranked higher than the term “adolescents” both before and after the COVID-19 pandemic, and that the prevalence of both terms increased after the pandemic. The onset of OCD in early childhood tends to be more refractory than that in adolescence (37). Elucidating the mechanisms underlying the early-onset intractability of OCD in children remains an urgent research priority. It is important to note that PANDAS can induce OCD, and that this may affect the large number of OCD studies conducted in children. Addressing whether the age of OCD onset has shifted toward younger individuals following the COVID-19 pandemic and elucidating the underlying biological mechanisms, should be the focus of future research.

Neural circuits have been recognized as central to the pathophysiology of OCD. In particular, the cortico-striato-thalamo-cortical (CSTC) loop plays a key pathological role OCD development (38, 39). The terms related to the basal ganglia, including the striatum, consistently ranked among the top 30 most prevalent keywords in molecular-focused OCD research both before and after the COVID-19 pandemic. This highlights the need for continued focus on these circuits. In addition, the increased prevalence of the term “basal ganglia” - which includes globus pallidus, substantia ngigra, ventral pallidum, subthalamic nucleus and striatum - after the COVID-19 pandemic may indicate that OCD research is expanding to encompass broader neural circuits. Furthermore, this expansion may be associated with the increased prevalence of the term “functional connectivity” after the pandemic.

Although OCD is not a type of depressive disorder, selective serotonin reuptake inhibitors (SSRIs) are currently used as first-line treatment for OCD. However, higher doses than those typically used for depression are often required to achieve therapeutic effects in OCD (40). This standard therapy is based on previous reports that OCD pathophysiology is distinctly associated with serotonin malfunction (41, 42). Interestingly, “serotonin” ranked lower in prevalence than “dopamine” before the COVID-19 pandemic, but rose in rank thereafter. The reason for this change remains unclear, but may be associated with the need for caution when using anti-virus agents in combination with SSRIs or dopamine suppression (43). Alternatively, the increased prevalence of the term “serotonin” may reflect a growing demand for therapeutic strategies targeting OCD, particularly in the context of worsening OCD phenotypes during the COVID-19 pandemic.

In addition to brain regions, neural circuits, and neurotransmitters, the contributions of different cell types to OCD should also be noted. Approximately 50% of the brain is made up of cells other than nerve cells (44). Microglia are divided into resting and activated microglia, which are reported to be related to immunity and inflammation (45). Indeed, activated microglia may be associated with the pathophysiology of OCD in human (46). Reports have also raised the possibility of a link between astrocytes and OCD (47, 48). This study found that only “adult microglia” was present in the research mapping before the COVID-19 pandemic. Elucidating the pathophysiology of OCD by focusing on the physiological roles of these non-neuronal cells could become an important research topic in the future.

The present study has several limitations. First, bibliometric analysis relies on quantitative indicators such as the number of citations, frequently occurring keywords, and network analysis, and it is not possible to directly evaluate the quality of research impact of papers. In addition, the number of publications and citations associated with a topic does not necessarily correspond to important findings within that topic. Second, this study does not directly link bibliometric trends to clinical outcomes. Moreover, while the study maps visualize related-keywords, their relevance or impact is not validated. Although the present molecularly focused analysis may help inform emerging hypotheses regarding the pathophysiology of OCD, further research is needed to clarify how these molecular targets can be translated into diagnostic frameworks or novel therapeutic strategies. Finally, it is important to note that there is often a time lag between when research is conducted and when it is published. Some studies published after the onset of the COVID-19 pandemic may have been conducted beforehand. Therefore, the observed trends may reflect not only the impact of the pandemic but also pre-existing research trends that are unrelated to COVID-19. Nevertheless, our findings indicate that OCD research may be entering a stage of more comprehensive and multifaceted stage that considers its diversity.

## Conclusions

This bibliometric analysis demonstrated that molecular-focused OCD research has evolved over the past decade with an increasing focus on gene expression research, meta-analysis, and overlapping with other mental disorders. The COVID-19 pandemic may have influenced these trends, and recent studies have focused on inflammation-related pathways, suggesting their potential involvement in the pathophysiology of OCD. Further research on novel molecular targets and interdisciplinary approaches to OCD are required.

## Data Availability

The raw data supporting the conclusions of this article will be made available by the authors, without undue reservation.
