# Supplementary figures and images for: Mapping research trends in obsessive-compulsive disorder before and after the COVID-19 pandemic: a bibliometric analysis focusing on its molecular mechanisms
Source: Front Psychiatry. 2025 Jul 2;16:1615497. doi: 10.3389/fpsyt.2025.1615497 (PMC12263677; doi:10.3389/fpsyt.2025.1615497)

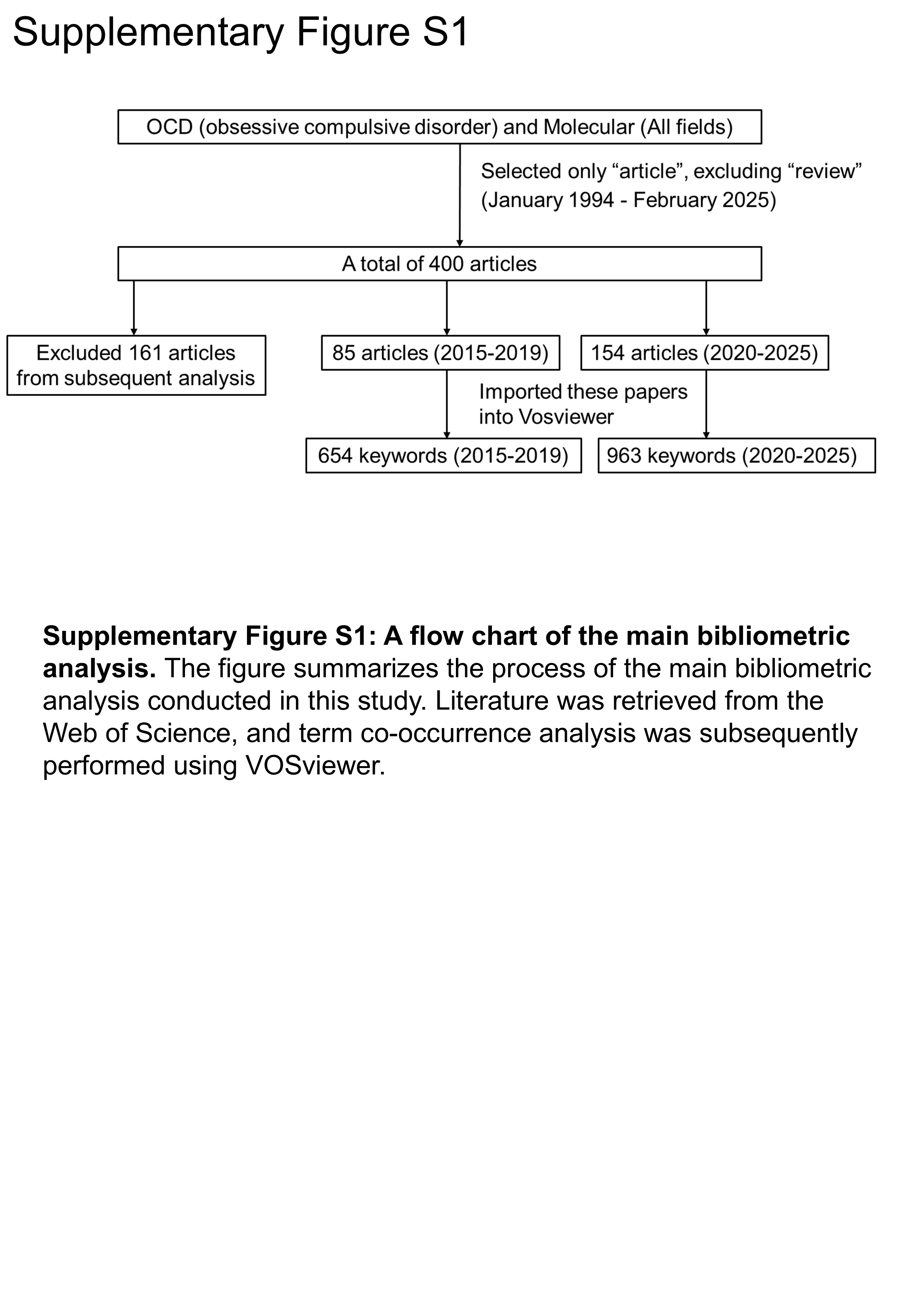

Supplement: Supplementary file 1 [file Image1.tif]
